# Giant oscillations in a triangular network of one-dimensional states in marginally twisted graphene

**DOI:** 10.1038/s41467-019-11971-7

**Published:** 2019-09-05

**Authors:** S. G. Xu, A. I. Berdyugin, P. Kumaravadivel, F. Guinea, R. Krishna Kumar, D. A. Bandurin, S. V. Morozov, W. Kuang, B. Tsim, S. Liu, J. H. Edgar, I. V. Grigorieva, V. I. Fal’ko, M. Kim, A. K. Geim

**Affiliations:** 1 School of Physics and Astronomy, University of Manchester, Manchester, M13 9PL UK; 2 National Graphene Institute, University of Manchester, Manchester, M13 9PL UK; 3 Institute of Microelectronics Technology and High Purity Materials, Russian Academy of Sciences, Chernogolovka, 142432 Russia; 4 The Tim Taylor Department of Chemical Engineering, Kansas State University, Manhattan, KS 66506 USA

**Keywords:** Materials science, Physics

## Abstract

At very small twist angles of ∼0.1°, bilayer graphene exhibits a strain-accompanied lattice reconstruction that results in submicron-size triangular domains with the standard, Bernal stacking. If the interlayer bias is applied to open an energy gap inside the domain regions making them insulating, such marginally twisted bilayer graphene is expected to remain conductive due to a triangular network of chiral one-dimensional states hosted by domain boundaries. Here we study electron transport through this helical network and report giant Aharonov-Bohm oscillations that reach in amplitude up to 50% of resistivity and persist to temperatures above 100 K. At liquid helium temperatures, the network exhibits another kind of oscillations that appear as a function of carrier density and are accompanied by a sign-changing Hall effect. The latter are attributed to consecutive population of the narrow minibands formed by the network of one-dimensional states inside the gap.

## Abstract (web summary)

The conductivity of marginally-twisted bilayer graphene is predicted to persist in presence of a bandgap-opening interlayer bias, owing to a network of 1D conductive states at domain boundaries. Here, the authors report Aharonov–Bohm oscillations up to 100 K, whereas at liquid helium temperatures another kind of oscillation appears, due to progressive population of the narrow minibands formed by the 2D network of 1D states inside the gap.

## Introduction

**Fig. 1:**
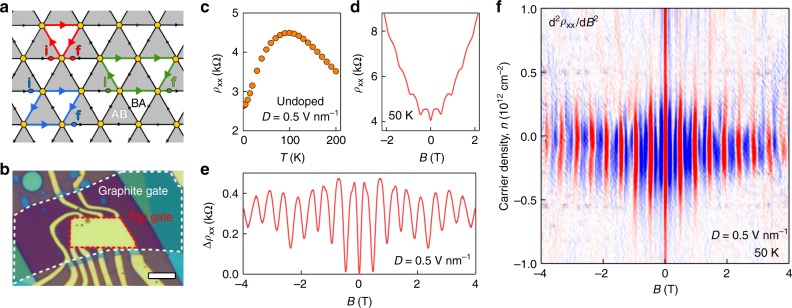
Aharonov–Bohm oscillations in marginally twisted bilayer graphene. **a** Schematic of MTG: Its superlattice forms a triangular network of AB/BA domain walls^11–13^. The white and gray areas denote domains with the Bernal stacking; yellow circles—regions with AA stacking. Black arrows show the propagation direction for electrons in one of the valleys; colored arrows are examples of trajectories encircling one (red), two (blue), and three (green) triangular domains, which are responsible for different frequencies in Aharonov–Bohm oscillations. For example, the 2nd harmonic arises from interference between electrons propagating along the trajectories indicated by blue arrows (starting points are marked by ‘**i**’; finishing by ‘**f**’). **b** Optical micrograph of one of the studied devices. The graphite and top gates are indicated by color-coded dashed curves. The bright yellow regions are Cr/Au contacts to graphene and the top gate. Scale bar, 5 µm. The device exhibited carrier mobility of ∼10^4^ cm^2 ^V^−1^ s^−1^ at *n* $\approx$10^12^ cm^−2^, which is typical for small twist angles^12,16^ where irregularity in positions of domain walls probably causes additional scattering. **c** Temperature dependence of undoped MTG with the energy gap of ∼50 meV induced by interlayer bias. **d** Magnetoresistance at the NP for the same device at 50 K. **e** Same as **d** but a monotonic background is subtracted for clarity. **f** Same Aharonov–Bohm oscillations for different doping. Instead of subtracting the magnetoresistance for each *n* as in **e**, we plot ${\mathrm{d}}^2\rho _{{\mathrm{xx}}}(B)/{\mathrm{d}}B^2$ which removes the smooth background without shifting positions of oscillations’ extrema. Blue-to-red scale, ±4 kOhm T^−2^

The electronic properties of graphene superlattices have attracted intense interest^1–4^ that was further stimulated by the recent observation of novel many-body states in twisted bilayer graphene (BLG)^2,5–18^. The latter system exhibits qualitative changes with varying the twist angle $\theta$ between the two graphene layers, which is caused by subtle interplay between the interlayer electron hybridization and the periodic crystallographic pattern known as a moiré superlattice^2,5–18^. For small $\theta$, the superlattice period is given by $\lambda = a/[2\sin \left( {\theta /2} \right)] \approx a/\theta$ and is much longer than graphene’s lattice constant *a*. The recent interest in twisted BLG has been focused on so-called magic angles (typically, close to $\theta \approx 1.1$°) at which the low-energy superlattice minibands become almost flat^5,6^ promoting electron-electron correlation effects and leading to unconventional insulating and superconducting states^8–10^. At the marginal twist angles, $\theta \ll 1^\circ$, the electronic structure is expected to become qualitatively different from that formed at magic or larger $\theta$ because the BLG superlattice undergoes a strain-accompanied lattice reconstruction such that there appear large (submicron) triangular domains with alternating Bernal (AB and BA) stacking order^11–13^. The domain regions are rather similar to the conventional BLG and, if the displacement field *D* is applied between the layers, a sizeable energy gap $\delta$ opens in the spectrum^11,19,20^, making the domains insulating^19^. Under these conditions, marginally twisted graphene (MTG) bilayers may still remain electrically conductive because walls between AB and BA domains allow one-dimensional (1D) chiral states^11–18,21–24^ (Fig. 1a). For an AB/BA domain wall, there are four (2 spins and 2 valleys) gapless 1D states on each side. They propagate in opposite directions for different valleys and split apart at the superlattice’s vertices where the stacking changes into AA (Fig. 1a). The unit block for this 2D network is an equilateral triangle with the area $A = \frac{{\sqrt 3 }}{4}\lambda ^2$, half the size of the superlattice unit cell that includes both AB and BA domains.

In this Communication, we study the electronic properties of MTG and report exceptionally strong Aharonov–Bohm oscillations^25^ arising from electron interference along the triangular loops forming the chiral network. Yet another type of oscillations is observed in MTG’s resistivity as a function of carrier density, which indicates the presence of multiple electronic minibands inside the gap. Our work shows that marginally twisted BLG is markedly distinct from other 2D electronic systems, including BLG at larger twist angles, and offers a fascinating venue for further research.

## Results

### Experimental devices

Our devices were made from MTG that was prepared following the procedures developed in ref. ^26^. In short, a monolayer graphene crystal was teared into two parts that were placed on top of each other by parallel transfer accompanied by small rotation. In our case the rotation angle $\theta$ was set close to zero (nominally, 0 to 0.1°). Subsequent transport measurements (see below) showed that the resulting bilayers exhibited twist angles of ≤0.25°. The MTG crystals were encapsulated in hexagonal boron nitride to improve their electronic quality and, using lithography techniques, shaped into dual-gated Hall bar devices such as shown Fig. 1b (Supplementary Note 1). The top gate was the standard metal-film electrode whereas the bottom gate was thin graphite, which further improved devices’ electronic quality and reduced charge inhomogeneity. Four MTG devices were studied in detail, all exhibiting similar behavior. Below we focus on the results obtained for a device with $\theta \approx$ 0.10° (Supplementary Note 2) which had the highest uniformity, as witnessed from practically the same magnetotransport characteristics observed using different contact configurations. For completeness, examples of the behavior observed for other MTG devices are provided in Supplementary Note 3.

### 1D conductive network

To study electron transport through the expected network created by AB/BA domain walls, we applied the displacement field *D* using the top and bottom gates (Supplementary Note 1), which opened an energy gap in the Bernal-stacked regions^11,19,20^. For *D* *=* 0.5 V nm^−1^ (achievable without a risk of damaging the devices), the gap $\delta$ inside AB and BA regions should be^20^ ∼50 meV so that they become highly insulating at temperatures *T* ≤ 50 K as known from the experiments on standard BLG^19^. In contrast, our MTG devices exhibited a distinctly metallic behavior such that longitudinal resistivity *ρ*_xx_ decreased with decreasing *T*, reaching a few kOhms at liquid-helium *T* (Fig. 1c). This shows that, at the charge neutrality point (NP), MTG hosts a metallic system (Supplementary Note 5), in contrast to Bernal-stacked BLG and in agreement with the expected 1D transport along AB/BA walls. At *T* > 100 K, the temperature behavior changed so that *ρ*_xx_ decreased with increasing *T* (Fig. 1c). The latter observation is attributed to thermally activated carriers in the gapped AB and BA regions, similar to the case of standard BLG^19^. The insulating behavior could also be suppressed by field-effect doping. For *D* *=* 0.5 V nm^−1^, it typically required carrier densities *n* above ±3–5 × 10^11^ cm^−2^ to start populating the conduction and valence bands, as seen from a drop in *ρ*_xx_ (Supplementary Note 4).

### Aharonov–Bohm oscillations

**Fig. 2:**
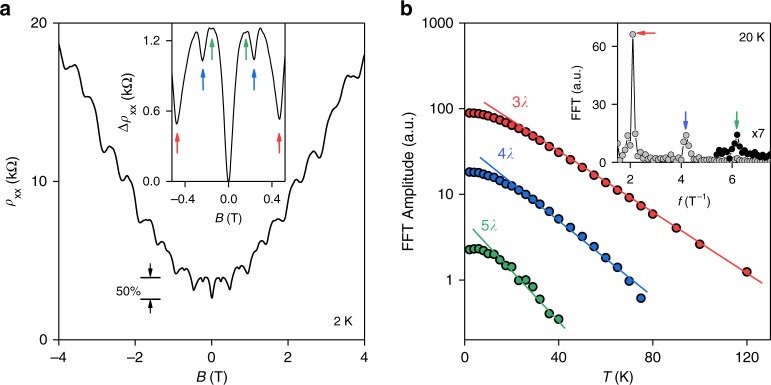
High-order harmonics in the Aharonov–Bohm oscillations. **a** Typical magnetoresistance curves near the main NP (*T* *=* 2 K, *D* = 0.5 V nm^−1^). Insert: Zoom-in close to zero *B*. The arrows mark the minima that correspond to $\phi _0$ piercing one (red), two (blue) and three (green) triangular domains shown in Fig. 1a. **b** Temperature dependence for the three oscillation frequencies (symbols). Solid lines: Guides to the eye indicate that the slopes for the 2nd and 3rd harmonics are, respectively, ∼4/3 and 5/3 times steeper than for the main frequency. Insert: Example of our fast-Fourier-transform (FFT) analysis (gray circles). The peaks are marked using the same color coding as in **a**. The third-harmonic peak is magnified for clarity (black symbols)

The network of conducting AB/BA walls revealed itself most clearly in strong magneto-oscillations that were periodic in magnetic field *B* and observed for all our MTG devices. Examples are shown in Fig. 1d–f and Supplementary Fig. 2c. The oscillations developed with increasing *D* and persisted until AB and BA domains became conductive because of either doping or temperature. For example, at *D* *=* 0.5 V nm^−1^, this meant |*n*| up to 5 × 10^11^ cm^−2^ and *T* up to 120 K as seen in Fig. 1f and Fig. 2, respectively. The periodic-in-*B* oscillations persisted up to several Tesla where they became overwhelmed by Shubnikov-de Haas oscillations (Supplementary Note 3). We attribute the low-*B* oscillations to the Aharonov–Bohm effect^25^ for electrons propagating along the triangular network of 1D channels hosted by AB/BA walls. Indeed, interference between electronic states propagating along, e.g., the red loop in Fig. 1a is expected to vary periodically with the magnetic flux piercing the domain area *A*. For the particular device in Fig. 1, we found the oscillation period ${\mathrm{\Delta }}B \approx$ 0.48 ± 0.02 T which translates into one flux quantum $\phi _0 = h/e$ per $A \approx$ 0.86 ± 0.04 × 10^−10^ cm^2^ and yields $\lambda \approx$ 140 nm or $\theta \approx$ 0.10° ± 3%, in agreement with the area found from the position of superlattice NPs (Supplementary Note 2).

As *T* decreased below 50 K, the Aharonov–Bohm oscillations grew exponentially, reaching more than 1 kOhm in amplitude at liquid-helium *T*, that is, nearly ∼50% of zero-*B* resistivity (Fig. 2a). Furthermore, higher frequency harmonics became visible at low *T* (inset of Fig. 2a). For quantitative analysis, *ρ*_xx_(*B*) curves for a given *T* were Fourier-transformed (see the inset of Fig. 2b). The peaks in the Fourier plot reveal the main periodicity ${\mathrm{\Delta }}B \approx$ 0.48 T (same as in Fig. 1) plus two fractional periods, ${\mathrm{\Delta }}B$/2 and ${\mathrm{\Delta }}B$/3. The latter correspond to twice and thrice larger areas involved in the interference pattern and can be attributed to the loops such as those indicated by the blue and green arrows in Fig. 1a. This assignment agrees well with the fact that the higher harmonics decayed notably faster with increasing *T* than the main-frequency oscillations (Fig. 2b), as expected because of the larger circumferences of the blue and green loops. Moreover, the suppression of Aharonov–Bohm oscillations is usually described by the dependence^27^ exp(−*L*/*L*_ϕ_) where *L* is the length of the interference loops, and *L*_ϕ_(*T*) is the decoherence length. The color-coded lines in Fig. 2b show that the decay rates for the 1st, 2nd, and 3rd frequencies followed the ratios *L*/*L*_ϕ_ expected from the circumferences of the three involved loops (*L* *=* 3*λ*, 4*λ*, and 5*λ*, respectively) for a given *L*_ϕ_(*T*).

### Minibands inside the gap

**Fig. 3:**
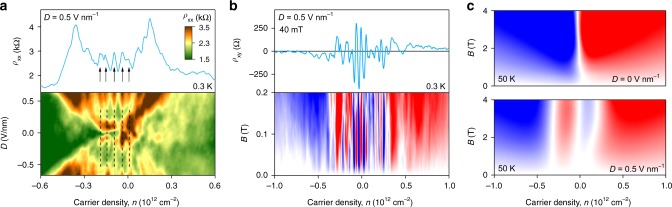
Minibands in the gapped state of marginally twisted BLG. **a** Longitudinal resistivity as a function of *n* and *D* at 0.3 K. Upper panel: Crosscut of the map at *D* *=* +0.5 V nm^−1^. **b** Same oscillatory behavior in Hall resistivity as a function of *n* and *B* for the given *D*. Color scale: ±250 Ohm. Upper panel: Crosscut at *B* = 40 mT. The arrows in **a** mark some of the maxima in *ρ*_xx_ which correspond to NPs (zero *ρ*_xy_) in **b**. **c**, Same as **b** but measured at 50 K. Upper and lower panels are for *D* = 0 and 0.5 V nm^−1^, respectively. Color scale: ±3 kOhm

We also studied the network’s transport properties as a function of doping, keeping *n* sufficiently low to remain in the insulating state for the AB and BA domains. Figure 3 shows that our MTG devices exhibited strong oscillations in their zero-*B* resistivity as a function of *n*. This oscillatory behavior was not due to mesoscopic (interference) fluctuations^28^: It remained the same for measurements using different contact configurations and different *D*. The oscillations were even more profound in Hall resistivity *ρ*_xy_(*n*) that reversed its sign many times within the gapped region (Fig. 3b and Supplementary Fig. 2b). The observed behavior can be understood as changes in the global interference pattern with varying *n* and, hence, the Fermi wavelength. This is conceptually similar to Aharonov–Bohm oscillations that are caused by the periodic-in-*B* phase modulation. The expected main periodicity $\Delta n$ is given^7,8,16^ by 4/*A* which corresponds to one extra electron per AB or BA domain, taking into account the fourfold degeneracy. Figure 3a, b yield characteristic $\Delta n \approx$ 5 ± 0.5 × 10^10^ cm^−2^ and, hence, $A \approx$ 0.80 ± 0.09 × 10^−10^ cm^2^, which agrees well with the area 0.86 ± 0.04 × 10^−10^ cm^2^ found from the main period of Aharonov–Bohm oscillations. The observed oscillations in *n* can equally be interpreted as the consecutive filling of electronic minibands formed by the triangular 2D lattice of 1D chiral states as suggested in refs. ^14,15^ (also, see Supplementary Note 5). The electronic spectrum for each such miniband has both electron and hole like states, which should lead to multiple NPs and sign-changing *ρ*_xy_(*n*). Accompanying oscillations in *ρ*_xx_(*n*) are also expected to appear as the minibands are filled one by one. The number of the observed minibands can be estimated by counting the number of NPs (for example, in Fig. 3b there are about 10 oscillations inside the gapped region (|*n*| < 3 × 10^11^ cm^−2^). As *D* = 0.5 V nm^−1^ results in $\delta \approx$ 50 meV^20^, the average width *ε* of those minibands is ∼5 meV.

## Discussion

The reason why the minibands evolve periodically in *B* (Aharonov–Bohm oscillations) but not fully so in *n* could be the following. First, the interlayer bias *D* and finite *n* remove the degeneracy between AB and BA domains so that 1D states propagating at the opposite sides of AB/BA walls acquire somewhat different Fermi velocities^29^. This should lead to beatings between interference oscillations arising from AB and BA domains. Interference along the longer loops should further contribute to some randomness in the oscillatory behavior. To elucidate the mentioned analogy between oscillations caused by changing the Fermi wavelength and the miniband description, we estimate the energy difference $\Delta E$ = *hv*_DW_/*L* between the states arising from consecutive standing waves in the smallest loop *L* = 3*λ* where *h* is the Planck constant and *v*_DW_ is the drift velocity of the 1D states at AB/BA walls. After taking into account the lifted degeneracy between AB and BA domains, the experimental value for $\Delta E$ is 2*ε* $\approx$ 10 meV, which yields *v*_DW_ $\approx$ 10^6^ m s^−1^, comparable to graphene’s Dirac velocity. Such a large value of *v*_DW_ suggests extremely sharp boundaries between AB and BA domains (Supplementary Note 6), which agrees with the strain reconstruction for small-*θ* superlattices as found by electron microscopy^12^ and infrared nano-imaging^13^.

Finally, let us point out some other surprising features of electron transport through the AB/BA domain network. First, the oscillations caused by filling the minibands are rapidly smeared by *T* and completely disappear above 20 K in both *ρ*_xx_ and *ρ*_xy_ (Supplementary Note 4 and Fig. 3c). This is probably expected as the minibands are only 5 meV apart and, therefore, cannot be resolved at such high *T*. In contrast, the Aharonov–Bohm oscillations survive to much higher *T* (Fig. 2). On one hand, this is perhaps not surprising because of relatively long *L*_ϕ_ typical for graphene. On the other hand, the robustness seemingly contradicts to the narrow minibands description. To explain this conundrum, we refer to Brown-Zak oscillations^30^ that also have their origins in the Aharonov–Bohm effect but appear for superlattices with 2D conductivity rather than in a network of conductive 1D states. Brown–Zak oscillations in graphene superlattices were found to be exceptionally robust and survived above room *T*, despite the thermal smearing covered many minibands^30^. This is because the minibands respond to *B* in a uniform manner, which was described in terms of changing the average group velocity^30^. A similar description is likely to be applicable to the narrow minibands in MTG and explain the robust Aharonov–Bohm oscillations for the thermally smeared minibands.

Another puzzle is the reversal of the average Hall effect found for our conductive network. This is shown in Fig. 3c where *ρ*_xy_ is plotted for *D* = 0 and 0.5 V nm^−1^ at 50 K. Without an interlayer bias (top panel), the Hall response is normal, with positive *ρ*_xy_ for electrons and negative for holes (for a given *B* direction). In contrast, as the triangular network was formed by applying the interlayer bias, the Hall effect reversed its sign. The normal behavior recovered only at high *n*, inside the conduction and valence bands. The reversal of the average sign of *ρ*_xy_ implies that the network’s minibands are predominantly hole-like for electron doping and vice versa for hole doping. This observation does not follow from any of the existing models^14,15^. To understand the reversal qualitatively, we evoke an analogy with 1D chiral states in the quantum Hall effect. From a semiclassical perspective, these states can be viewed as skipping orbits and, along the inner boundaries of AB and BA domains, electrons would then circulate in the direction opposite to that for cyclotron orbits, that is, an average effect from such small closed loops would be hole-like. Accordingly, a collection of skipping orbits on a triangular network may result in a hole-like Hall effect. This analogy requires theoretical corroboration in terms of minibands’ electronic spectra of MTG.

## Methods

### Device fabrication

Our MTG samples were encapsulated between two hBN crystals and prepared using the tear-and-stack method (see Supplementary Note 1 for more details). We used few-layer graphite as the back gate to reduce charge inhomogeneity. The final devices were shaped into the Hall bar geometry using electron-beam lithography and reactive-ion etching.

### Electrical measurements

The measurements were carried out using the standard low-frequency lock-in technique with excitation frequencies of 6 to 30 Hz and currents of typically 10 nA at 0.3 K and 100 nA at higher temperatures.

## Supplementary information

Supplementary Information

## Data Availability

The data that support the findings of this study are available from the corresponding author upon reasonable request.
