# Supplementary material for: Giant oscillations in a triangular network of one-dimensional states in marginally twisted graphene
Source: Nat Commun. 2019 Sep 5;10:4008. doi: 10.1038/s41467-019-11971-7 (PMC6728432; doi:10.1038/s41467-019-11971-7)
Supplement: Supplementary file 1 — Supplementary Information [file 41467_2019_11971_MOESM1_ESM.pdf]

SUPPLEMENTARY INFORMATION

Giant oscillations in a triangular network of one-dimensional states in marginally twisted graphene

S. G. Xu *et. al.*

Supplementary Note 1

Device fabrication and electrical measurements. The studied MTG devices were assembled by the standard dry-transfer[1,2] and tear-and-stack[3,4] techniques as briefly explained below. First, we chose a crystal of hexagonal boron nitride (hBN) that would later serve as the top gate dielectric. The crystal was picked up using a double-layer polymer film that consisted of a thin layer of polypropylene carbonate (PPC) spun onto a polydimethylsiloxane (PDMS) film. Then we used a precision micromanipulator to place this hBN crystal on top of a graphene monolayer (prepared on an oxidized Si substrate) so that hBN covered approximately half of the graphene crystal (Supplementary Figure 1a). Next we slowly peeled the hBN crystal (attached to graphene) off the substrate, which resulted in the graphene crystal teared into two parts. The part remaining on the substrate was rotated by up to 0.1° and then picked up by the graphene-hBN stack. This resulted in MTG on the hBN substrate (right panel of Supplementary Figure 1a). The substrate temperature was kept at ~ 40 °C to reduce thermally-induced strain and, also, to avoid possible spurious rotations induced by annealing. Particular care was taken to avoid any contact between MTG and PPC, which allowed a clean interface between the two graphene layers. Finally, an hBN crystal for the bottom gate dielectric was selected and picked up using the same procedures. The resulting four-layer stack was then released onto a graphite crystal residing on an oxidized Si wafer. This graphite crystal served as a bottom gate electrode. We used regions of the MTG bilayer, which lied outside the graphite gate, to define quasi-1D contacts using the etching recipe reported previously[5]. This was followed by the deposition of Cr (3 nm) and Au (60 nm) to make metallic contacts. Further electron-beam lithography and metal deposition were employed to define the top gate electrode. The latter also served as an etch mask for the final plasma etching and, to this end, had a Hall bar configuration that was accordingly projected onto MTG.

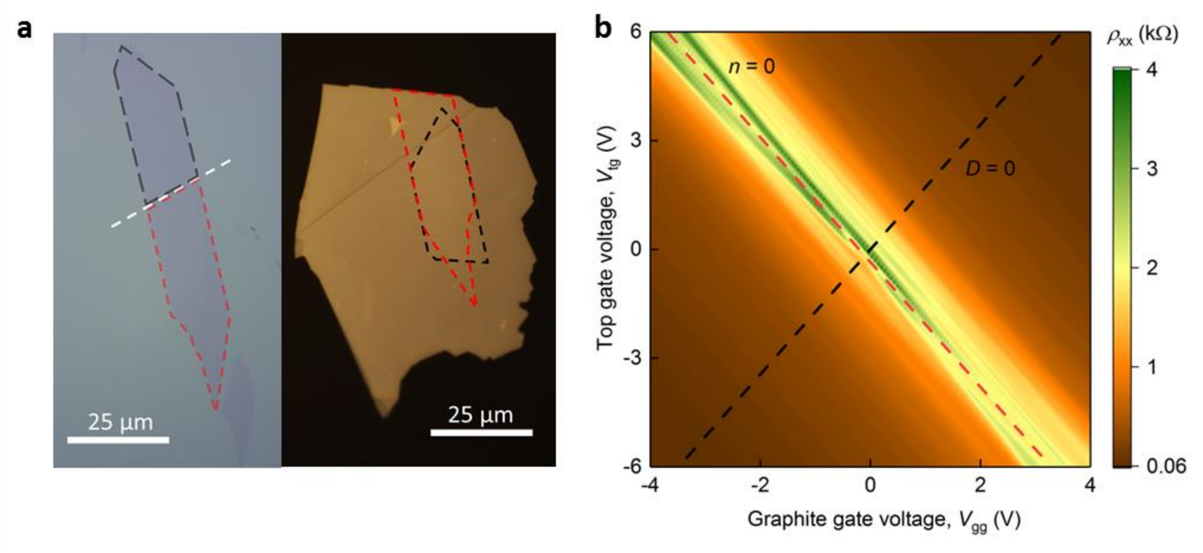

Supplementary Figure 1 | Tear-and-stack assembly of marginally twisted graphene bilayers and dual-gate characterization. a, Optical images illustrating MTG assembly. Left panel: Initial graphene on an oxidized Si wafer. The white line indicates where the tear occurred later. Right panel: Resulting twisted bilayer graphene attached to the top-gate hBN crystal. The black and red dashed curves outline the two teared parts of graphene crystals in both images. **b,** Typical map of $\rho_{xx}(V_{tg}, V_{gg})$ for MTG devices at 2 K. The red and black lines show the conditions for zero n and D, respectively. By changing gate voltages to move parallel to these lines allowed measurements under constant n or D.

The measurements were carried out using the standard low-frequency lock-in techniques with excitation currents below 100 nA, which made heating and nonlinear effects negligible. Most of the data presented in the main text have been taken under the constant displacement field *D*. The dual gated devices allowed us to control its value independently of the total carrier density *n*. The latter is the sum of the densities induced by the top and the bottom gates: $n = \frac{1}{e}(C_{tg}V_{tg} + C_{gg}V_{gg})$, where e is the electron charge, V_{tg} and V_{gg} are the top and graphite gate voltages, and C_{tg} and C_{gg} are the top and graphite gate capacitances per unit area, respectively. The capacitances were found independently through Hall measurements. The displacement field was calculated as $D = \frac{1}{2\varepsilon_0}(C_{tg}V_{tg} - C_{gg}V_{gg})$, where ε_0 is the vacuum permittivity. To fix *D*, we applied V_{tg} and V_{gg} as illustrated in Supplementary Figure 1b by changing both gate voltages, which allowed us to vary the carrier density *n* at a constant *D*.

Supplementary Note 2

Determining twist angles. The actual twist angle θ between the two graphene layers was determined using two independent methods. First, we used $\rho_{xx}(n)$ and $\rho_{xy}(n)$ measurements to find additional neutrality points (NPs) arising due to the superlattice potential. At zero *D*, they were very close to the main NP, on the steep slopes of the peak in ρ_{xx} (see Supplementary Figure 2a), and we found this procedure unreliable for our devices with extremely small angles. To overcome the difficulty, we applied a finite displacement field, which led to clear NPs in both ρ_{xx} and ρ_{xy} as seen in Fig. 3 and Supplementary Figure 2. This can be attributed to the development of a highly insulating state for the Bernal-stacked AB and BA regions so that they no longer shunted electron transport along AB/BA walls. The higher the displacement field the larger the gap and, hence, more minibands could fit inside.

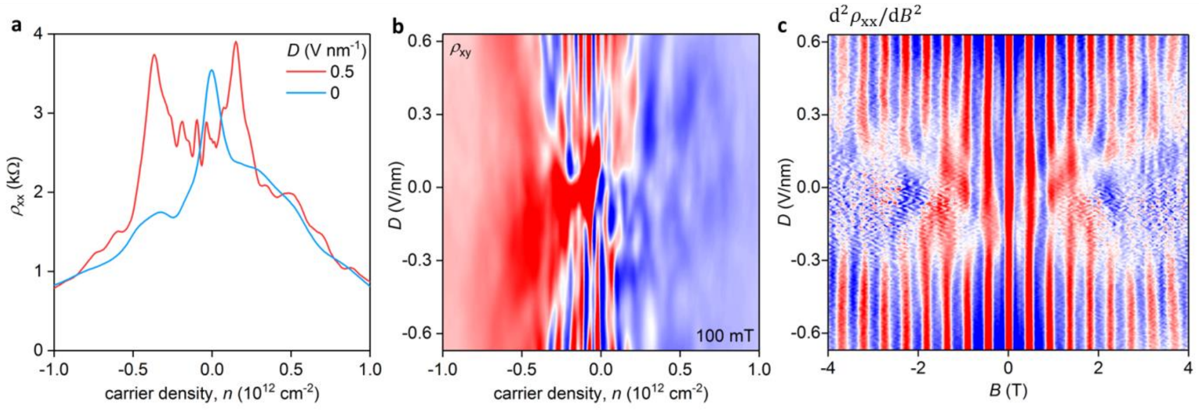

Supplementary Figure 2 | Multiple neutrality points in the gapped state. a, ρ_{xx} measured for two displacement fields *D* = 0 and 0.5 V nm^{-1} at 3 K. **b**, $\rho_{xy}(n, D)$ at *B* = 100 mT and *T* = 2 K. Blue-to-red scale, ±250 Ohm. **c**, Second derivative of $\rho_{xx}(B)$ as a function of *D* at 50 K and zero doping. Blue-to-red scale, ±4 kOhm T^{-2}.

As discussed in the main text, the area A of the triangular AB and BA domains is given by $A = 4/\Delta n$ where Δn is the distance between the neutrality points. This distance can be found from either ρ_{xx} or ρ_{xy} measurements. For the known area A, it is straightforward to find the twist angle

$$\theta = 2\arcsin(\sqrt{\frac{\sqrt{3}a^2}{16A}})$$

(see equations for the lattice and superlattice periods in the main text). For the devices discussed in the main text, $\Delta n = 5 \pm 0.5 \times 10^{10}$ cm^{-2} and the above formula yields the twist angle of 0.104° ±0.006°. Another way to determine the twist angle is from the periodicity ΔB of Aharonov-Bohm oscillations (Fig. 2b and Supplementary Figure 2b). In this approach, the domain area is given by $A = \phi_0/\Delta B$, and from the oscillation period 0.48 ± 0.02 T in Fig. 2b of the main text, we find θ = 0.10 ±0.002°, in agreement with the above estimate using Δn. Similarly good agreement was found for the other devices. Indeed, for MTG in Supplementary Figure 3a (top panel), $\Delta B = 2.7 \pm 0.2$ T yielded θ = 0.235±0.01° whereas its $\Delta n = 28 \pm 4 \times 10^{10}$ cm^{-2} yielded θ = 0.245±0.025°, and the device in Supplementary Figure 3a (bottom) showed $\Delta B = 0.85 \pm 0.05$ T and $\Delta n = 8 \pm 1 \times 10^{10}$ cm^{-2} yielding θ = 0.133±0.04° and 0.131±0.08°, respectively.

Note that the Aharonov-Bohm oscillations became better developed above a certain displacement field (Supplementary Figure 2c), which is attributed to the fact that 1D electron transport along AB/BA domain walls was no longer electrically shortened by a finite 2D conductivity of the Bernal-stacked regions.

Supplementary Note 3

Further examples of Aharonov-Bohm oscillations. We fabricated several MTG devices, four of which showing highest homogeneity were studied in detail. All four exhibited pronounced Aharonov-Bohm oscillations under large displacement *D*, and Supplementary Figure 3 shows examples of the magneto-oscillations for two other devices with twist angles of ~0.13˚ and ~0.24˚. At liquid-helium *T* and in low *B*, both devices showed the first and second harmonics of Aharonov-Bohm oscillations. As the field increased, the Aharonov-Bohm oscillations became overwhelmed by Shubnikov-de Haas oscillations.

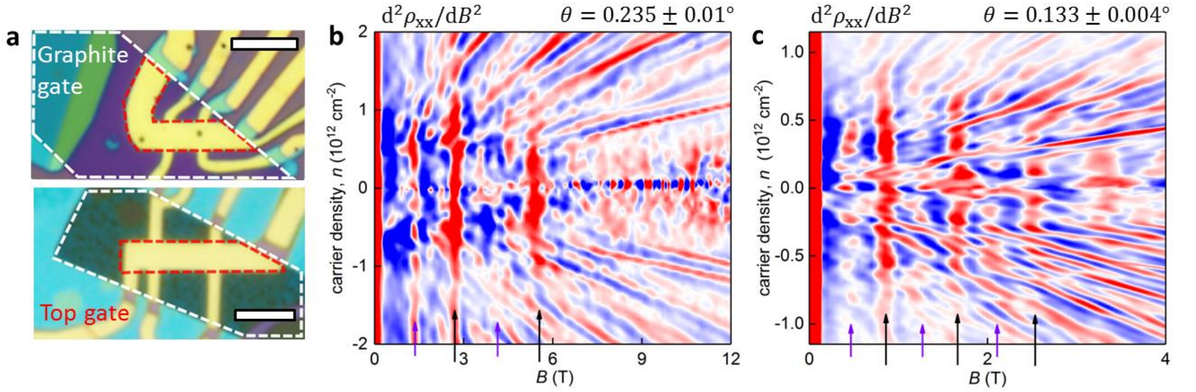

Supplementary Figure 3 | Aharonov-Bohm oscillations for different twist angles. a, Optical micrographs of two other devices with $\theta \approx 0.24˚$ (top) and 0.13˚ (bottom). Scale bars, 5 µm. **b** and **c,** Second derivative of $\rho_{xx}(B)$ for the MTG in the top and bottom images of (a), respectively. The measurements were carried out at *T* = 2 K and *D* = 0.4 and 0.1 V nm^{-1}, respectively. Blue-to-red scale: ±6 and ±30 kOhm T^{-2} for (b) and (c), respectively. The black (purple) arrows mark maxima for the first (second) harmonic.

Supplementary Note 4

Temperature dependence in the gapped state. Despite the large energy gaps δ were induced by the interlayer bias *D*, all our MTG devices exhibited the metallic behavior at low *n* inside the expected gapped state (Fig. 1c of the main text). This is further corroborated in Supplementary Figure 4 that shows $\rho_{xx}(n)$ for *D* = 0.5 V nm^{-1} at different *T*. Although δ was ~ 50 meV in the Bernal-stacked

regions, ρ_{xx} increased with increasing T for n where Aharonov-Bohm oscillations were observed. This density range is indicated by the two dashed lines in Supplementary Figure 4. The figure shows that the additional resistance peaks due to the formation of narrow bands inside the gap became smeared at T of about 20 K. Nonetheless, electron transport due to the in-gap minibands remains seen up to 50 K. This is because charge carriers in the conduction and valence bands led to a lower rate of increase in $\rho_{xx}(T)$, which effectively resulted in the two sharp resistance peaks that can be attributed to the edges of the conduction and valence bands (see the red curve in Supplementary Figure 4). Only at T higher than ~100 K, 2D conductivity inside the Bernal-stacking regions overwhelmed the transport contribution from AB/BA walls, which led to the recovery of the behavior typical for Bernal bilayer graphene[6] with a monotonic decrease in ρ_{xx} with increasing T at low n due to thermally activated carriers. The observed behavior strongly supports the discussed concept of 1D states propagating along AB/BA walls.

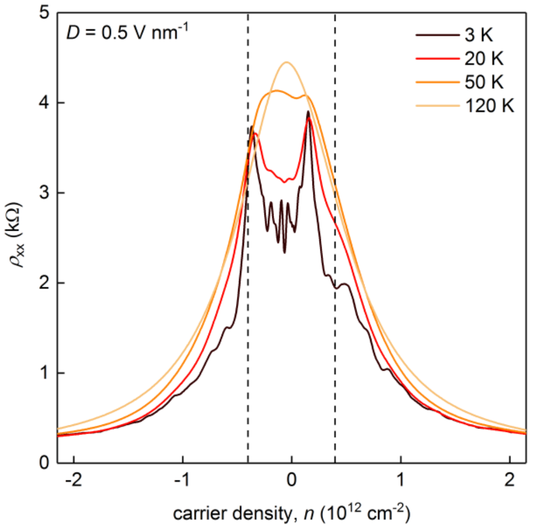

Supplementary Figure 4 | Resistivity as a function of carrier density at several characteristic temperatures. $\rho_{xx}(n)$ was measured at constant D = 0.5 V nm^{-1}. The vertical dashed lines indicate the approximate range where Aharonov-Bohm oscillations were observed in this device.

Supplementary Note 5

Miniband spectrum of the triangular network. For twisted bilayer graphene, recent theoretical efforts have focused mainly on its properties close to the main magic angle ($\theta \approx 1°$) where the two graphene sheets are assumed to be only rotated with respect to each other and their crystal lattices unperturbed[7–9]. This model is not applicable for marginal angles, $\theta \ll 1°$, where the two graphene layers become considerably strained[10]. The resulting lattice reconstruction was shown to create a rather macroscopic lattice of BLG domains with the conventional staking order and alternating AB and BA regions (Supplementary Figure 5a). The domains are separated by atomically-sharp walls[10].

For such a bilayer system, the interlayer bias opens the standard energy gap δ within the interior (Bernal-stacked) regions but also gives rise to 1D states propagating along AB/BA domain walls[11] (Supplementary Figure 5). The triangular metallic network can be described by a secondary, miniband spectrum that appears within the energy gap[12]. Supplementary Figure 6a shows this spectrum calculated using the model of ref.[12] where the minibands appear periodically in energy. The model[12] does not take into account changes in the Fermi velocity, which are generally expected

to occur near the gap edges (Supplementary Note 6). The latter effect leads to a departure from the periodic behavior, as illustrated in Supplementary Figure 5c.

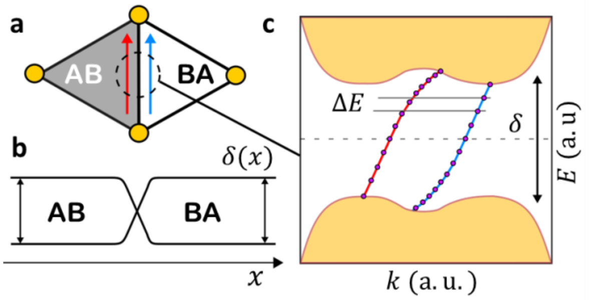

Supplementary Figure 5 | 1D chiral states at AB/BA domain walls. a, Schematic of MTG. White and grey areas: AB and BA domains with the Bernal stacking; yellow circles - regions with AA stacking. The red and blue arrows denote 1D states co-propagating on the opposite sides of the domain wall. **b**, Schematic of the gap inversion near the wall between AB and BA domains. **c**, Schematic of the domain wall's spectrum[16]. The plot shows that as we approach the conduction or valence bands the states on the opposite sides of the domain wall [same color coding as in (a)] acquire different Fermi velocities and, therefore, the corresponding minibands have different spacing (see Supplementary section 6).

Experimentally, minibands inside the gap should result in multiple peaks in resistivity ρ_{xx} which occur each time the Fermi level moves from one miniband to another crossing the Dirac-like neutrality point where the density of states goes to zero (Supplementary Figure 6b). Also, Hall resistivity ρ_{xy} is expected change its sign. This should happen twice more often than the peaks in ρ_{xx} because the effective mass changes its sign not only at the neutrality points but also at van Hove singularities (Supplementary Figure 6b). The described behaviour is in good agreement with that observed our experiments and shown in Fig. 3 and Supplementary Figure 2.

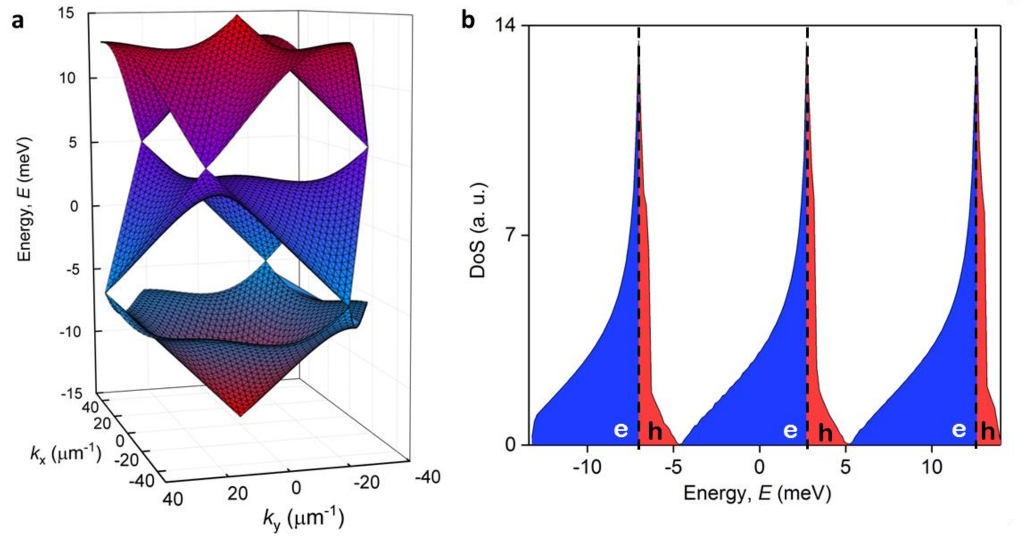

Supplementary Figure 6 | Miniband spectrum for the triangular network of 1D states. a, Spectrum calculated for θ = 0.1° using the model of ref.[12] with the scattering parameter α = 1.1. **b**, Density of states for the spectrum in (a). The vertical dashed lines indicate van Hove singularities, whereas the blue and red colored areas mark spectral regions with electron- and hole- like carriers, respectively.

Supplementary Note 6

Fermi velocity of 1D states at the domain walls. Recent theoretical studies[11,13–16] confirmed experimentally[17–19] have shown that a domain wall (DW) between oppositely gapped BLG regions (using oppositely oriented *D*), or at the interface between equally gapped AB and BA domains supports two co-propagating chiral 1D states inside the gap for each valley in graphene's band structure (Supplementary Figures 5,6). These states have an almost linear 1D dispersion and propagate in the opposite directions in the two valleys. Electron transport due to these 1D states is protected topologically, unless there are defects that generate intervalley scattering. For a sharp DW having a width $w < v/\sqrt{\gamma_1 \delta}$ (where γ_1 is the interlayer hopping), the drift velocity v_{DW} of the 1D states should be of the order of the Dirac velocity v in monolayer graphene, although the exact value is expected to depend on DWs' crystallographic orientation. The co-propagating 1D states are expected[16] to have very close values of v_{DW}, however those diverge for the energies away from the center of the BLG gap and approaching the band edges, as illustrated in Supplementary Figure 5c. Overlaying the two slightly different spectra with a typical energy spacing $\Delta E = v_{\mathrm{DW}} h/3\lambda$ (see the main text) results in a twice denser spectrum with the spacing $\varepsilon \approx v_{\mathrm{DW}} h/6\lambda$. Moreover, differences in the level spacing should lead to beatings in minibands' spectral manifestation (this is noticeable in the data shown in Fig. 3 of the main text). Because each of the standing waves gives rise a different moiré miniband, for a sharp domain wall we expect that the above ε determines the number of minibands

$$M \approx \delta/(\frac{v_{\mathrm{DW}} h}{6\lambda})$$

which fit inside the gap δ opened in the Bernal-stacked domains for a given *D*. By counting the number of minibands revealed by the ρ_{xx} and ρ_{xy} oscillations in Fig. 3, we obtained an experimental estimate for the drift velocity as $v_{\mathrm{DW}} \approx v$. Note that, for wider DWs such that $w \gg v/\sqrt{\gamma_1 \delta}$, the 1D states become slower and additional non-topological channels with parabolic dispersions appear. The cumulative effect of the latter two trends should result in a larger number of minibands inside the gap, which can be estimated as $\delta\gamma_1 (h\lambda/v)^2$. In the latter case, v_{DW} would be much smaller if estimated from the number of the experimentally observed NPs using the above equation. Therefore, the fact that the estimated v_{DW} is close to v confirms the general assumption that the lattice reconstruction in marginally twisted BLG transforms it into a network of narrow domain walls between relatively large AB and BA domains with the Bernal stacking.

Supplementary References

1. Kretinin, A. V. *et al.* Electronic properties of graphene encapsulated with different two-dimensional atomic crystals. *Nano Lett.* **14,** 3270–3276 (2014).
2. Wang, L. el. al. One-dimensional electrical contact to a two-dimensional material. *Science* **342,** 614–617 (2013).
3. Kim, K. *et al.* van der Waals heterostructures with high accuracy rotational alignment. *Nano Lett.* **16,** 1989–1995 (2016).
4. Cao, Y. *et al.* Superlattice-induced insulating states and valley-protected orbits in twisted bilayer graphene. *Phys. Rev. Lett.* **117,** 116804 (2016).
5. Ben Shalom, M. *et al.* Quantum oscillations of the critical current and high-field superconducting proximity in ballistic graphene. *Nat. Phys.* **12,** 318–322 (2016).
6. Oostinga, J. B., Heersche, H. B., Liu, X., Morpurgo, A. F. & Vandersypen, L. M. K. Gate-induced insulating state in bilayer graphene devices. *Nat. Mater.* **7,** 151–157 (2008).

7. Suárez Morell, E., Correa, J. D., Vargas, P., Pacheco, M. & Barticevic, Z. Flat bands in slightly twisted bilayer graphene: Tight-binding calculations. *Phys. Rev. B* **82,** 121407(R) (2010).
8. Bistritzer, R. & MacDonald, A. H. Moiré bands in twisted double-layer graphene. *Proc. Natl. Acad. Sci.* **108,** 12233–12237 (2011).
9. Angeli, M., Tosatti, E. & Fabrizio, M. Valley Jahn-Teller effect in twisted bilayer graphene. *arXiv:1904.06301v2* (2019).
10. Yoo, H. *et al.* Atomic and electronic reconstruction at the van der Waals interface in twisted bilayer graphene. *Nat. Mater.* **18,** 448–453 (2019).
11. Martin, I., Blanter, Y. M. & Morpurgo, A. F. Topological confinement in bilayer graphene. *Phys. Rev. Lett.* **100,** 036804 (2008).
12. Efimkin, D. K. & Macdonald, A. H. Helical network model for twisted bilayer graphene. *Phys. Rev. B* **98,** 35404 (2018).
13. Zarenia, M., Pereira, J. M., Farias, G. A. & Peeters, F. M. Chiral states in bilayer graphene: Magnetic field dependence and gap opening. *Phys. Rev. B - Condens. Matter Mater. Phys.* **84,** 125451 (2011).
14. Cosma, D. A. & Fal'Ko, V. I. Trigonal warping effect on velocity and transverse confinement length of topologically confined states in bilayer graphene. *Phys. Rev. B - Condens. Matter Mater. Phys.* **92,** 165412 (2015).
15. Pelc, M., Jaskólski, W., Ayuela, A. & Chico, L. Topologically confined states at corrugations of gated bilayer graphene. *Phys. Rev. B - Condens. Matter Mater. Phys.* **92,** 085433 (2015).
16. Lane, T. L. M. *et al.* Ballistic electron channels including weakly protected topological states in delaminated bilayer graphene. *Phys. Rev. B* **97,** 045301 (2018).
17. Li, J. *et al.* Gate-controlled topological conducting channels in bilayer graphene. *Nat. Nanotechnol.* **11,** 1060–1065 (2016).
18. Ju, L. *et al.* Topological valley transport at bilayer graphene domain walls. *Nature* **520,** 650–655 (2015).
19. Yin, L. J., Jiang, H., Qiao, J. Bin & He, L. Direct imaging of topological edge states at a bilayer graphene domain wall. *Nat. Commun.* **7,** 11760 (2016).
